# Tracking global resources and capacity for health research: time to reassess strategies and investment decisions

**DOI:** 10.1186/s12961-023-00979-7

**Published:** 2023-09-11

**Authors:** Taghreed Adam, Ambinintsoa H. Ralaidovy, Anna Laura Ross, John C. Reeder, Soumya Swaminathan

**Affiliations:** Science Division, World Health Organization, Geneva, Switzerland

**Keywords:** Health research and development, Priority setting, Funding, Investments, Low- and middle-income countries, Global

## Abstract

The COVID-19 pandemic and more recently the Monkeypox outbreak emphasize the urgency and importance of improving the availability and equitable distribution of resources for health research across rich and poor countries. Discussions about the persistent imbalances in resource allocation for health research between rich and poor countries are not new, but little or no progress has been made in redressing these imbalances over the years. This is critical not only for emergency preparedness, but for the worlds’ ability to improve population health in an equitable manner. Concerned with the lack of progress in this area, Member States of the World Health Organization requested the establishment of a Global Observatory on Health Research and Development, with the aim of consolidating, monitoring and analyzing relevant information on health research and development, with a view to informing the coordination and prioritization of new investments. In this commentary, we highlight some of the striking disparities from the Observatory’s analysis over the 5 years since its establishment and reflect on what is needed to overturn stagnant progress.

## Background

Among the insights that the COVID-19 pandemic has offered the world is the realization that it is possible for the global community to unite in mobilizing resources and technical expertise to accelerate the development of needed health technologies. But COVID-19 pandemic also emphasized how complicated it is to ensure equitable distribution and access to these lifesaving technologies across rich and poor countries [1].

Concerns about the persistent imbalance in resource allocation for health research between rich and poor countries have been voiced repeatedly over the past decades, with little progress observed despite various recommendations and targets being set to monitor and inspire progress [1–6]. High-income countries (HICs) continue to produce a disproportionately large proportion of research publications (close to 80%) compared to the percentage of the global population living in these countries (16%) and the disease burden (disability-adjusted life years) affecting them (10%), focusing mainly on diseases corresponding to their needs, with relatively less attention given to the diseases and needs of lower income countries [7]. Upper-middle-income countries (UMICs) come next with 17% of the total number of publications, while low-income countries (LICs) produce only 0.6% [7]. These findings illustrate the persistence of the established (1999) “10/90 gap”, which estimated that less than 10% of the world’s resources for health research are spent on the major health problems of 90% of the world’s population living in low- and middle-income countries [8].

This lack of progress in aligning research investments to population health needs is claiming millions of lives and results in considerable economic suffering each year. Tuberculosis (TB) for example kills approximately one person every 17 s —around 1.5 million in 2021 alone [9]. This is a huge toll of lives lost and suffering from one single disease that predominantly affects the poor. Despite high-level commitments to raise sufficient resources to end the global TB epidemic by 2035, resource allocation is not meeting the required investments to achieve the targets of the “end TB” strategy [10]. Of the required US$2 billion per year to accelerate the development of new diagnostics, therapeutic strategies, and vaccines, only US$ 1 billion was available in 2021 [11].

In recognition of these persistent inequalities, Member States of the World Health Organization requested the establishment of a Global Observatory on Health Research and Development (hereafter called the Observatory) to inform the coordination and prioritization of new investments in health research and development (R&D) globally [12, 13].

The Observatory was launched in January 2017. It builds on existing and newly data to provide a comprehensive assessment of the health research and development landscape by answering the questions of who is doing what, where, with what resources and where the research capacities lie. While its initial focus was primarily on research and product development for diseases of the poor, as new resources became available it was able to gradually expand its scope to now cover all health conditions and all types of health research.

For the fifth year since its establishment, the Observatory continues to identify striking discrepancies in resource allocation and capacity for conducting health research and product development across countries, with little to no improvements in global targets for health R&D over this period. The objective of this commentary is to highlight some of the discrepancies from the Observatory’s analysis (see Box 1 for a summary) and to reflect on what is needed to overturn the stagnant progress.

The findings highlighted in this commentary focus on four areas: the distribution of grant funding for health research; availability of health researchers; availability and distribution of higher education institutions with disciplines relevant to health research; and the extent to which global targets for health R&D are met. In each case, reference to the full analysis, interactive data visualization, and other supporting information such as the data sources, scope and methods of analysis and limitations are provided. While the focus here is on selected findings, a wealth of information and numerous possibilities for tailored investigation of the data can be explored through the Observatory’s online platform with over 26 interactive data visualizations to date and a growing resource of databases and resources [12, 14, 15].

Box 1 Highlights from 5 years of monitoring global resources and capacity for health research

- No evidence of research investments aligning better to public health needs. Analysis of grants for health research by 10 of the world’s largest international funders of health research in 2020 shows that:
  - A very small share of all grant funding went to low-income countries (0.2%)
  - As little as 0.2% of all grant funding for noncommunicable diseases (NCDs) was allocated to institutions in low- and middle-income countries, where an estimated 7 out of 10 NCD deaths now occur
  - Funding for WHO priority areas such as the diseases on the WHO list of neglected tropical diseases^1^ remains low (0.6% of all grants funding)
- Significant disparities in human resource capacity for health research persist between countries, with high-income countries having approximately 56 times more health researchers (in full time equivalent terms) per million inhabitants than the low-income countries
- Wide gaps in the availability of higher education institutions. The median number of higher education institutions per million people in low-income countries is 0.81 compared to 3.78, 3.16, 1.64 in high, upper-middle and lower-middle income countries, respectively
- No evidence of tangible progress in meeting global targets for health R&D such as those related to official development assistance allocated to medical research and the share of gross domestic expenditures on health R&D

Source: Global Observatory on Health Research and Development [12]

## Key findings

I. There is large inequity in distribution of grants for health research across countries and diseases
  Analysis of investments on grants by 10 of the world’s largest international funders of health research that report data to World RePORT online database shows significant differences in resource allocation across countries [13]. In 2020, out of grants totaling US$ 37 billion, low-income countries (LICs) received only 0.2% (US$ 85 million). Lower-middle-income countries (LMICs) and upper-middle-income countries (UMICs) received each 0.5% (US$ 188 million US$ 193 million, respectively. These figures are very similar to the shares and amounts awarded by these funders four years earlier. Out of a total of US$ 32.8 billion awarded in 2016, 0.1% (US$ 37 million), 0.4% (US$ 114 million) and 0.7% (US$ 240 million) of all grants were awarded to LICs, LMICs and UMICs in turn [16].
  With respect to grant funding by topic, the analysis shows no evidence of alignment between grant distribution decisions and global priorities or the evolving epidemiological profiles of low- and middle-income countries. For example, as little as 0.2% (US$ 48 million) of an approximate total of US$ 21.4 billion of grant funding for noncommunicable diseases (NCDs) was allocated to institutions in low- and middle-income countries, where an estimated 7 out of 10 NCD deaths occur [17]. Similarly, a very small proportion of all grant funding targeted a WHO priority area such as the designated neglected tropical diseases^1^ (approx. 0.6%) or R&D Blueprint pathogens^2^ (approx. 4%) [17]. Analysis of grant funding for the four previous years (2016–2019) are also available from the Observatory and show similar figures to those presented here [13]. (See links to previous versions at the bottom of the page.)
II. The distribution of health researchers shows huge disparity between high- and low-income countries
  The density of health researchers per population continues to show striking disparities across countries. Based on the most recent publicly available data from 82 countries, HICs have approximately 56 times more health researchers (in full time equivalent terms) per million inhabitants (391) than the low-income group of countries (7), ranging from 1,158 per million in Singapore to 0.2 per million in Zimbabwe [18].
  Based on available data from 74 (out of 82) countries, although female researchers account for 51% (weighted average) of the health researchers globally, the proportion ranges from approximately 53% in HICs and UMICs, 49% in LMICs, to only 24% in LICs. This gap has widened in recent years (4 years earlier it was 51% in HICs and 27% in LICs) [18] (see links to previous years’ analyses at the bottom of the webpage).
III. Wide gaps between high- and low-income countries in the availability of higher education institutions and opportunities for research training
  Large disparities across countries also exist in the availability and distribution of higher education institutions and research training opportunities [19]. Based on data from 178 countries available from the World Higher Education Database, in 2019, LICs had the lowest median number of higher education institutions per million people (0.81) compared to the other three income groups (3.78, 3.16, 1.64 in HICs, UMICs and LMICs, respectively).
  The number of institutions that offer the opportunity for research training linked to obtaining an advanced postgraduate degree also varies widely across countries in the different income groups—at one extreme is Madagascar (LIC), where none of the 25 higher education institutions offer this training, compared with 1461 (80.5%) of the 1814 higher education institutions of the United States of America (HIC) that do so.
IV. No evidence of tangible progress in meeting global targets for health R&D across all income groups
  The Observatory tracks two global targets for health R&D: the gross domestic expenditure on health R&D (health GERD) as percent of gross domestic product (GDP); and the percent of official development assistance for health (health ODA) allocated to medical research.
  Health GERD as % of GDP is compared to four benchmarks (0.05%; 0.10%; 0.15% and 0.20% of GDP) following the targets recommended by the 2012 Consultative Expert Working Group Report: the target for developed countries was 0.15–0.2% of their GDP and for developing countries with potential research capacity, 0.05–0.1% [5].
  Based on the most recent data available from 86 countries, many did not meet the target for their income group, although a few exceeded it. Over half (49 countries) across all four income groups did not reach the minimum target of investing at least 0.05% of GDP on health GERD. Out of 30 HICs, only 11 met or exceeded their target (i.e., investing at least 0·15–0·20% of GDP on health GERD) [20].
  The target used to assess the share of health ODA allocated to medical research is 5%, set out in the 1990 report of the Commission on Health Research for Development [4]. Using data from 133 ODA-recipient countries which reported to OECD for 2020, well less than 5% of health ODA had been allocated to medical research in the majority of countries, and some received no ODA for medical research. Apart from Gambia and Uganda (two LICs), all recipient countries where the 5% target was met are UMICs [21].

## Concluding thoughts and reflections

The findings from the Observatory’s analysis to date emphasize the striking disparities in resource allocation for health research between rich and poor countries, and between diseases of the rich and those of the poor. Moreover, the funding gaps have not improved over time, doing little to narrow the wide gaps in the availability of health researchers and post-graduate research training opportunities between high-income and low- and middle-income countries.

While many of these finding are not surprising, it is sobering to see that they paint a very similar picture to that described by the authors of the 1990 report of the Commission on Health Research and Development [4]. Resource allocation decision-making around health R&D has not been able to redress these imbalances over the past three decades. The various indicators and targets that have been proposed over the past decades to track progress have not encouraged changes in decision-making strategies that would ensure progress towards the targets. Moreover, many countries still do not systematically collect the data needed to track them.

With the steady emergence of public health emergencies of international concern, most recently with the Monkeypox outbreak, it is critical to step-up actions at all levels to resolve this reality [1]. The current global efforts to review and monitor countries’ pandemic preparedness response and their capacity to conduct locally relevant research offer an opportunity to re-visit needs across the whole health R&D space to generate the progress that has been lacking [22, 23]. This is an opportunity that should not be missed: to encourage more countries to routinely report on the proposed indicators, to perhaps review and revise some of the more long-standing indicators, and to stimulate countries to translate global agreements on R&D indicators into their resource-allocation decisions. Funders of health R&D (of all types) also have an important role in generating progress: through coordinated research prioritization decisions considering global and local (something that is consistent with what the “decolonizing global health movement” is calling for) public health needs, and by enabling informed decisions and coordinated efforts through increased data sharing of their funding activities [24].

This commentary illustrates the value of tracking and analysing health R&D in a regular and comprehensive manner to be able to highlight both progress and the lack thereof. Health R&D data sharing has improved over the past few years, but several limitations still exist, particularly on availability of data from LMICs. More data sharing, particularly on R&D investments and capacity, is needed to enable better coordination and informed decisions. The Global Observatory on Health R&D can assist in these efforts by continuing to serve as the comprehensive platform to track and analyze relevant health R&D data and document progress in key indicators over time. However, action must come from these data if we are to capitalize on the global cooperation driven by the COVID-19 pandemic and start to make gains on the vision of a more equitable global health future.

## Data Availability

Data and analysis presented in this commentary are available on the Global Observatory on Health Research and Development website: https://www.who.int/observatories/global-observatory-on-health-research-and-development. Future analysis, updates, and expansions of the analysis in this study will be available on the Observatory.

## Abbreviations

GDP: Gross domestic product
HICs: High-income countries
UMICs: Upper-middle-income countries
LICs: Low-income countries
Health GERD: Gross domestic R&D expenditures on health and medical sciences
NCD: Noncommunicable disease
ODA: Official development assistance
TB: Tuberculosis
WHO: World Health Organization

## Footnotes

1 https://www.who.int/health-topics/neglected-tropical-diseases

2 https://www.who.int/activities/prioritizing-diseases-for-research-and-development-in-emergency-contexts
